# Zinc stimulates glucose oxidation and glycemic control by modulating the insulin signaling pathway in human and mouse skeletal muscle cell lines

**DOI:** 10.1371/journal.pone.0191727

**Published:** 2018-01-26

**Authors:** Shaghayegh Norouzi, John Adulcikas, Sukhwinder Singh Sohal, Stephen Myers

**Affiliations:** College of Health and Medicine, School of Health Sciences, University of Tasmania, Newnham Campus, Launceston, Tasmania, Australia; China Medical University, TAIWAN

## Abstract

Zinc is a metal ion that is an essential cell signaling molecule. Highlighting this, zinc is an insulin mimetic, activating cellular pathways that regulate cellular homeostasis and physiological responses. Previous studies have linked dysfunctional zinc signaling with several disease states including cancer, obesity, cardiovascular disease and type 2 diabetes. The present study evaluated the insulin-like effects of zinc on cell signaling molecules including tyrosine, PRSA40, Akt, ERK1/2, SHP-2, GSK-3β and p38, and glucose oxidation in human and mouse skeletal muscle cells. Insulin and zinc independently led to the phosphorylation of these proteins over a 60-minute time course in both mouse and human skeletal muscle cells. Similarly, utilizing a protein array we identified that zinc could active the phosphorylation of p38, ERK1/2 and GSK-3B in human and ERK1/2 and GSK-3B in mouse skeletal muscle cells. Glucose oxidation assays were performed on skeletal muscle cells treated with insulin, zinc, or a combination of both and resulted in a significant induction of glucose consumption in mouse (p<0.01) and human (p<0.05) skeletal muscle cells when treated with zinc alone. Insulin, as expected, increased glucose oxidation in mouse (p<0.001) and human (0.001) skeletal muscle cells, however the combination of zinc and insulin did not augment glucose consumption in these cells. Zinc acts as an insulin mimetic, activating key molecules implicated in cell signaling to maintain glucose homeostasis in mouse and human skeletal muscle cells. Zinc is an important metal ion implicated in several biological processes. The role of zinc as an insulin memetic in activating key signaling molecules involved in glucose homeostasis could provide opportunities to utilize this ion therapeutically in treating disorders associated with dysfunctional zinc signaling.

## Introduction

Insulin resistance is a common pathophysiological condition in which patients present with perturbed biological responses to endogenous insulin leading to compromised glucose homeostasis specifically in liver and skeletal muscle [1]. The contribution of insulin resistance in various diseases such as type 2 diabetes (T2D), obesity, liver cirrhosis, atherosclerosis and cardiovascular disease [1, 2] is highly significant. A foremost concern for people with insulin resistance is the progressive failure of pancreatic β-cell function (a major determinant of type 2 diabetes progression) and compromised insulin secretion. Therefore, prevention strategies that take advantage of this “window of opportunity” (before β-cell failure) to prevent or lessen disease progression would have an enormous impact on the health and wellbeing of our communities. Currently, zinc is being investigated for its role in cell signaling pathways that are amendable to glucose homeostasis and thus have implications for insulin resistance and type 2 diabetes [3].

Zinc is present in all parts of the body including organs, tissues, fluids and secretions [4] and plays a critical role in a wide variety of biological processes [5, 6]. For example, zinc has a unique and extensive role in nucleic acid and lipid metabolism, cell signaling, growth and differentiation, apoptosis, enzyme activity, and brain and immune function [7]. Normal zinc homeostasis has a critical role in the release and action of insulin to maintain glucose homeostasis [8] since zinc has insulin mimetic activity and controls cellular processes including insulin receptor signal transduction, and insulin storage and secretion [9].

Zinc is essential for the processing, crystallization, and storage of insulin in pancreatic β-cells through the function of the pancreatic zinc transporter ZnT8 that moves zinc into insulin secretory cells [10, 11]. Beta-cell specific ZnT8 knock out mice display glucose intolerance, abnormal β-cell morphology, reduced islet insulin processing, a reduction in the total number of granules, and an increase in empty atypical granules suggesting that insulin crystallization and packaging is compromised [10]. In fact, there is a strong association between a mutation in ZnT8 (an arginine is replaced with a tryptophan at position 325 [R325W] in the cytoplasmic domain) which increases the risk of type 2 diabetes [12,13].

Early studies on zinc’s insulin mimetic activity revealed that this metal ion increased glucose metabolism in isolated rat adipocytes [14]. Similarly, in 3T3-L1 adipocyte cells, zinc treatment increased tyrosine phosphorylation of the insulin receptor β subunit and phosphorylation of Akt, and this was concomitant with enhanced glucose transport, independent of insulin [15]. These studies were further supported in C2C12 skeletal muscle cells where zinc could phosphorylate tyrosine, and the insulin receptor substrate 1 (ISR1) in the absence of insulin [16]. Moreover, zinc stimulated glucose consumption in normal and insulin-resistant L6 myotubes and was concomitant with the upregulation of Akt, the translocation of Glut4 and the phosphorylation of Gsk3β [17]. In delineating the mechanism whereby zinc exerts its effect on tyrosine phosphorylation, it was shown that zinc can increase tyrosine phosphorylation by inhibiting tyrosine phosphatases in C6 glioma cells [18,19]. These data are supported by recent studies that showed zinc ions inhibited PTP1B activity through direct binding to the enzyme [20]. These above studies on zinc as an insulin mimetic relay important information into the complexities of the role of this metal ion in signaling pathways and provide justification to further explore these pathways in more detail in metabolic processes associated with glycemic control.

Given the role of zinc as an insulin mimetic in controlling cellular metabolism, we sought to delineate the role of this metal ion in cellular processes amendable to controlling glucose homeostasis in skeletal muscle cells. We identified that in mouse and human skeletal muscle cells, zinc could affect several insulin-dependent cell signaling pathways that was concomitant with increased glucose oxidation.

## Materials and methods

### Antibodies

Akt (#9272), phospho-Akt (Ser473; #4058), phosphor-p44/42 MAPK (Erk1/2, Thr201/Tyr204; #8544), phospho-p44/42 MAPK (Eek1/2; #9102), SHP-2 (#3752), phospho-SHP-2 (Tyr580; #3703), phospho-Tyrosine (P-Tyr-100; #5465), GAPDH (#2118), and HRP-linked secondary antibodies (Anti-rabbit #7074; Anti-mouse #7067) were obtained from Cell Signaling Technology, USA. Intracellular signaling array kit (PathScan #7323) was also purchased from Cell Signaling Technology.

### Cell culture

Mouse C2C12 cells (a generous gift from Professor Steve Rattigan, Menzies Institute for Medical Research, Hobart, Australia) and human skeletal muscle cells (Thermo Fisher, Australia; catalog number A12555) were cultured in DMEM (Thermo Fisher) medium containing 10% fetal calf serum (FCS) and 100 U/mL penicillin/streptomycin (Thermo Fisher) and were maintained at 37°C and 5% CO_2_ in a humidified atmosphere. Cells were treated with insulin (10 nM), zinc (20 μM) and/or zinc pyrithione (10 μM) (Sigma Aldrich, Australia) for 60 minutes. The dose of zinc used is consistent with previous studies [21–23, 3] and represents the physiological level of plasma zinc which can range from 10.7 μM following a morning fast to 21.1 μM in healthy individuals [24].

Seventy-two hours before treatment, skeletal muscle cells were differentiated into myotubes by the addition of media containing 2% horse serum (Thermo Fisher). Three hours before treatments, the cells were exposed to serum-free conditions.

### Immunoblot analysis

Whole cell lysates were prepared in RIPA Lysis buffer in the presence of protease and protein phosphatase inhibitors (Thermo Fisher). Lysates were vortexed every 10 minutes for 1 hour at 4°C, and centrifuging at 15000 rpm for 5 minutes. Protein concentrations of the supernatants were determined using the BCA assay kit as per manufacturer’s instructions (Thermo Fisher). For western blotting analysis, equal amounts of proteins were heated to 95°C, separated on 4–15% SDS-polyacrylamide gels (Bio-Rad, Australia) and wet transferred to PVDF at 200 mV for 1 hour at 4°C (Polyvinylidene Difluoride) membranes (Thermo Fisher). The membrane was then blocked 2 hours in TBST (50 mmol/L Tris-Cl, pH 7.6, 150 mmol/L NaCl and 0.1% Tween 20) containing 5% (w/v) casein, and then incubated with the appropriate primary antibody as indicated overnight at 4°C. The membrane was washed four times with TBST, then incubated with HRP-conjugated secondary antibody for 1 hour. The membrane was again washed four times with TBST, and the blots were developed using SuperSignal West Femto kit [25] (Thermo Fisher). All phosho-immunoreactive species, Akt, ERK, SHP were normalized against total Akt, ERK or SHP. Immunoreactive phospho-tyrosine was normalized to GAPDH.

### Intracellular signaling array

The intracellular signaling protein array kit (chemiluminescent Readout) is a slide-based antibody array founded upon the sandwich immunoassay principle. The array kit allows for the simultaneous detection of 18 important signaling molecules when phosphorylated or cleaved. Skeletal muscle cells were treated with 10 nM insulin, 20 μM ZnSO_4_ in the presence of 10 μM sodium pyrithione (NaPy), 20 μM ZnSO_4_ alone, or no treatment (as a control) over 30 minutes and cells were lysed in RIPA lysis buffer supplemented with protease and protein phosphatase inhibitors. The assay procedure was performed according to the manufacturer’s protocol. Briefly, cell lysates were incubated on the array slide followed by a biotinylated detection antibody cocktail. Streptavidin-conjugated HRP and LumiGLO reagent were then used to visualize the bound detection antibody by chemiluminescence. An image of the slide was captured with a digital imaging system. Spot intensities for each phosho-immunoreactive protein in the insulin and zinc treated extracts were normalized to their untreated control. The image was analyzed, and the spot intensities quantified using densitometry array analysis software, Image J (https://imagej.nih.gov/ij/).

### Glucose oxidation assay

To determine the amount of glucose consumption by cells, glucose oxidase (GOx) activity assay kit was used as per manufacturer’s instructions (Sigma). To establish the glucose oxidation assay, cells were cultured in 96 well plate to reach 100% confluence. As the assay medium should be without any exogenous fuel substrate supplementation, cells were cultured in the serum-free media for another 24 hours. After 24 hours serum-free condition, cells were treated with 10 nM insulin, 20 μM of ZnSO_4_ in the presence of 10 μM NaPy, a combination of 10 nM insulin and 20 μM ZnSO_4_ in the presence of 10 μM pyrithione, and DMEM alone as a control. The concentration of glucose added to initiate glucose oxidation was 10 mM, which was determined in preliminary experiments to be above saturation. After incubation for 3 hours, cell lysate was collected and reaction mixture (GOx assay buffer, GOx developer, fluorescent peroxidase substrate and GOx substrate) was added and the absorbance was measured at 570 nm using a TECAN infinite M200 PRO flow cytometer. The background was corrected subtracting the blank measurement value from the sample measurement value.

### Insulin receptor inhibition

C2C12 skeletal muscle cells were treated with an insulin receptor tyrosine kinase inhibitor HNMPA-(AM)_3_ (abcam, Australia) at 0, 25, 50 and 100 μM for 1 hour followed by treatment of cells with either insulin (100 nM) for 30 mins, or zinc (20 μM) plus NaPy (10 μM) for 30 minutes. Following treatment, whole cell lysates were prepared and immunoblot analysis was performed on pAkt and total Akt as previously described in the methods above.

### Statistical analysis

Data, represented as the means ± SEM, were analyzed by the one-way ANOVA for multiple comparisons using the Graph Pad Prism 5 software to determine any significant differences. P < 0.05 was considered significant.

## Results

### Effect of zinc on insulin-dependent cell signaling molecules Akt, Tyrosine, SHP, ERK1/2, and PRAS40

**Fig 1:**
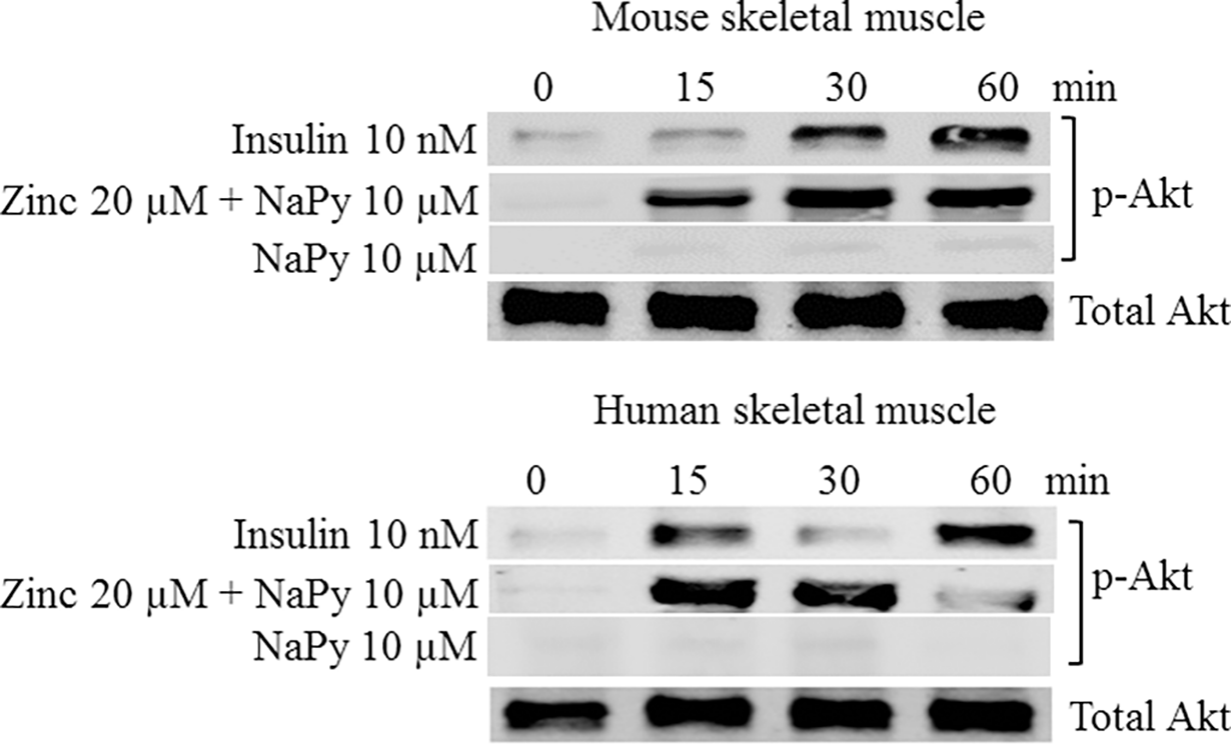
Analysis of p-Akt in mouse and human skeletal muscle cells treated with insulin, zinc, and NaPy over 60 min. Top and bottom panel represents mouse and human skeletal muscle cells, respectively. Time is shown in minutes from 0, 15, 30 and 60 and total Akt was used as an internal loading control in both panels and levels of pAKT were normalized to total Akt.

We measured the phosphorylation status of several proteins implicated in the insulin-signaling cascade in the presence of insulin or zinc. To assess that skeletal muscle cells responded to insulin we measured the phosphorylation status of protein kinase B (Akt), a well-established protein that is phosphorylated in the presence of insulin [26]. Skeletal muscle cells were treated with 10 nM insulin over 60 minutes and subsequent western blots were performed for the immunoreactivity of p-Akt. We observed that 10 nM of insulin-activated p-Akt in both C2C12 mouse and human skeletal muscle cells and thus confirmed our cells responded to insulin treatment (Fig 1). To test whether zinc has insulin mimetic activity to induce the phosphorylation of Akt, skeletal muscle cells were also treated with 20 μM ZnSO_4_ in the presence of 10 μM pyrithione. Similarly, zinc induced the phosphorylation of Akt within 15 minutes of treatment in both mouse and human cell lines (Fig 1).

**Fig 2:**
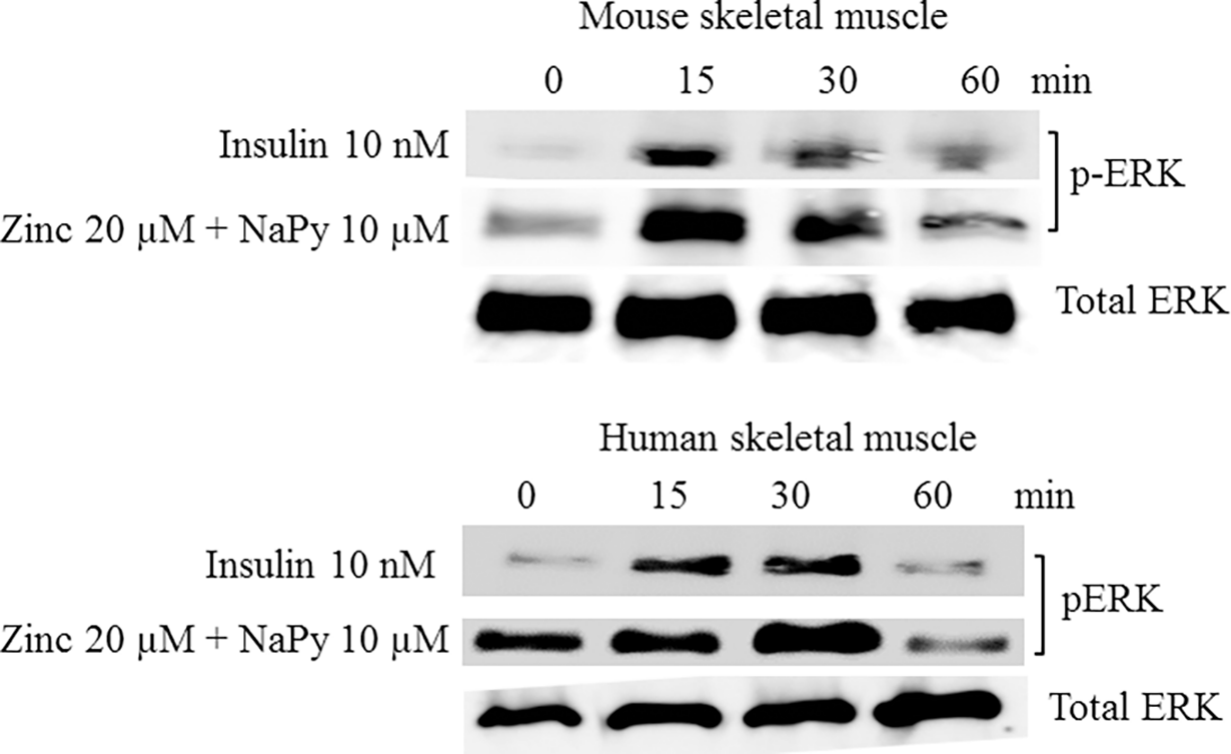
Analysis of p-ERK in mouse and human skeletal muscle cells treated with insulin, zinc, and NaPy over 60 min. Top and bottom panel represents mouse and human skeletal muscle cells, respectively. Time is shown in minutes from 0, 15, 30 and 60 and total ERK was used as an internal loading control in both panels and levels of p-ERK were normalized to total ERK.

The components of the mitogen-activated protein kinase/extracellular signal-regulated protein kinase (MAPK/ERK) pathway are modifiers of cellular insulin responsiveness [27], and therefore sensitive to insulin. Accordingly, we compared the phosphorylation status of ERK1/2 in cells treated with insulin and ZnSO_4_. Insulin induced the phosphorylation of ERK1/2 within 15 minutes of treatment in mouse and human skeletal muscle cells (Fig 2). Similarly, ZnSO_4_ in the presence of pyrithione induced ERK phosphorylation within 15 minutes of treatment in these cell lines (Fig 2).

**Fig 3:**
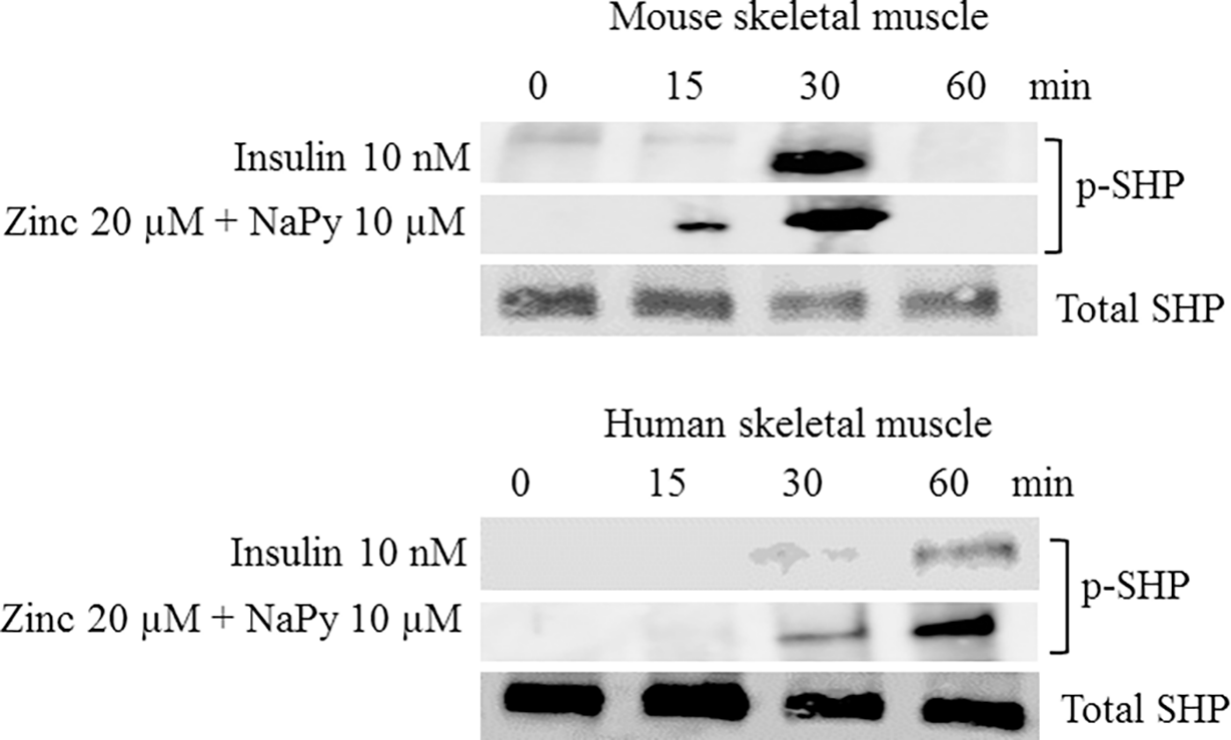
Analysis of p-SHP in mouse and human skeletal muscle cells treated with insulin, zinc, and NaPy over 60 min. Top and bottom panel represents mouse and human skeletal muscle cells, respectively. Time is shown in minutes from 0, 15, 30 and 60 and total SHP was used as an internal loading control in both panels and levels of p-SHP were normalized to total SHP.

We also identified that treatment of mouse and human skeletal muscle cells with insulin and zinc led to the phosphorylation of SHP-2, a protein tyrosine phosphatase involved in insulin signaling pathway [28] (Fig 3). Insulin and ZnSO_4_ induced pSHP-2 within 30 minutes of treatment in mouse and human skeletal muscle cells. pSHP-2 increased at 60 minutes of treatment in the human skeletal muscle cells, but not in mouse skeletal muscle (Fig 3).

**Fig 4:**
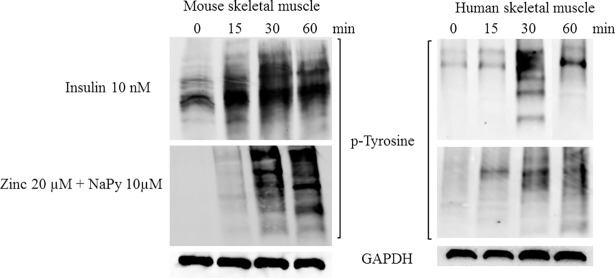
Analysis of p-tyrosine in mouse and human skeletal muscle cells treated with insulin, ZnSO_4_, and NaPy over 60 min. Top and bottom panel represents mouse and human skeletal muscle cells, respectively. Time is shown in minutes from 0, 15, 30 and 60 and GAPDH was used as an internal loading control in both panels and levels of p-tyrosine were normalized to GAPDH.

The activation and/or transduction of many signaling pathways including insulin signaling depends on the phosphorylation of tyrosine [29]. To test whether zinc plays a role in the phosphorylation of tyrosine residues in skeletal muscle, we treated mouse and human cells with insulin or ZnSO_4_ over 60 minutes. Insulin and ZnSO_4_ induced the phosphorylation of tyrosine residues over the time course in both mouse and human skeletal muscle cell lines (Fig 4).

**Fig 5:**
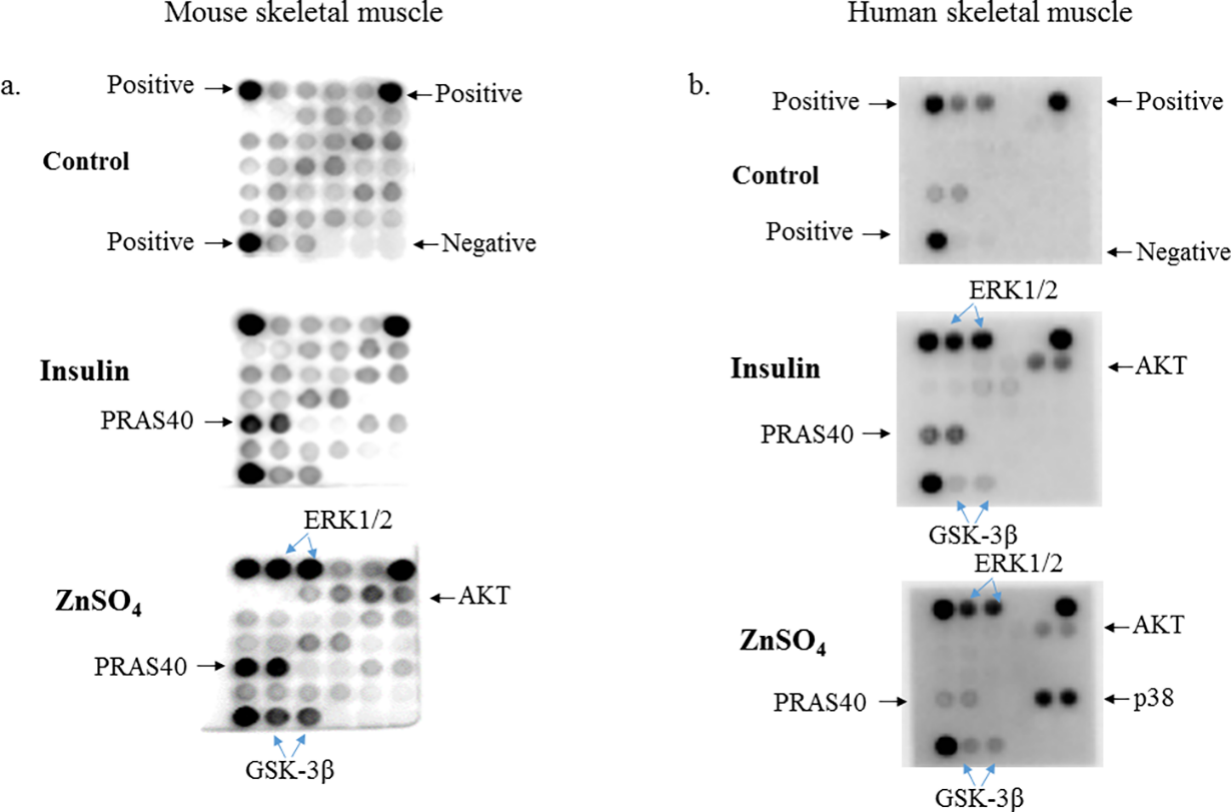
Effect of insulin and zinc on intracellular proteins via analysis of an intracellular protein array (see Table 1). a. Mouse skeletal muscle cells. Top panel, control (untreated cells), middle panel, insulin treated cells; bottom panel, ZnSO_4_ treated cells. Positive and negative controls are shown labelled on the control panel. Significant responses to insulin and ZnSO_4_ (PRAS40, Akt, ERK1/2 and GSK-3β) are shown. b. Human skeletal muscle cells. Top panel, control (untreated cells), middle panel, insulin treated cells; bottom panel, ZnSO_4_ treated cells. Positive and negative controls are shown labelled on the control panel. Significant responses to insulin (PRSA40, Akt and ERK1/2) and ZnSO_4_ (Akt, ERK1/2, GSK-3β and p38) are shown. All positive signaling molecules in the insulin and zinc treated arrays were normalized to their corresponding signaling molecule on the control array (no treatment).

We also detected significant changes in the phosphorylation of Akt, ERK1/2, glycogen synthesis kinase-3 (GSK-3β), Protein-Rich Akt Substrate of 40 kDa (PRAS40), and p38 using an Intracellular signaling protein array (Table 1 and Fig 5A and 5B). Positive signals for PRSA40, ERK1/2, Akt and GSK-3β were detected in skeletal muscle cells in the mouse protein array when compared to the control panel. Insulin activated PRAS40 and Akt, while ZnSO_4_ activated PRAS40, ERK1/2, Akt and GSK-3β in this system (Fig 5A). In the human protein array, we observed that insulin activated ERK1/2, Akt and PRAS40 when compared to the control. ZnSO_4_ activated ERK1/2, Akt, GSK-3β and p38 but not PRAS40 in this instance (Fig 5B).

**Table 1:** Protein target map of intracellular signaling array (cell signaling).

Positive control	ERK1/2	ERK1/2	Stat1	Stat1	Positive control
Stat3	Stat3	Akt (Thr308)	Akt (Thr308)	Akt (Ser473)	Akt (Ser473)
AMPKα	AMPKα	S6 Ribosomal Protein	S6 Ribosomal Protein	mTOR	mTOR
HSP27	HSP27	Bad	Bad	p70 S6 Kinase	p70 S6 Kinase
PRAS40	PRAS40	p53	p53	p38	p38
SAPK/JNK	SAPK/JNK	PARP	PARP	Caspase-3	Caspase-3
Positive control	GSK-3β	GSK-3β	Negative control	Negative control	Negative control

**Fig 6:**
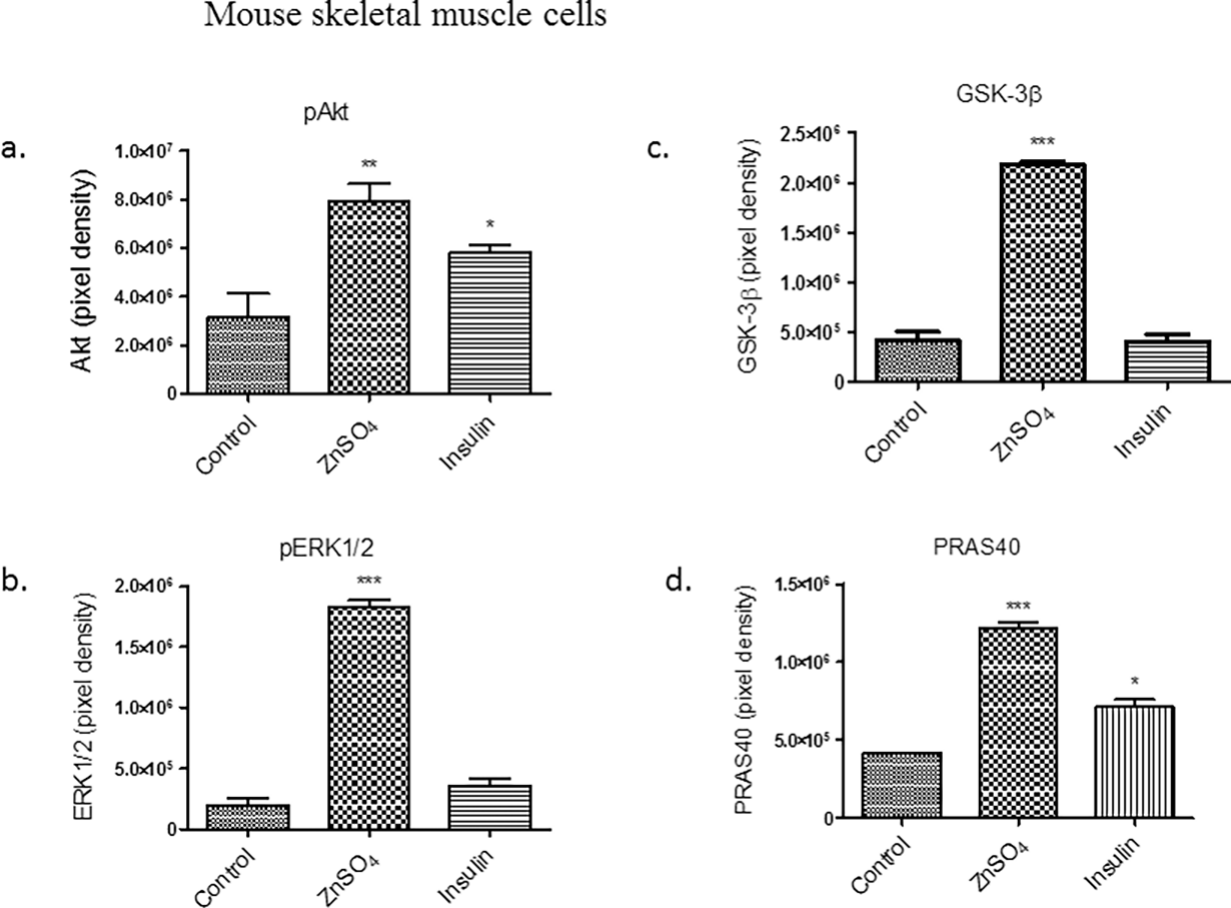
Densitometry results from Fig 5 protein array in mouse skeletal muscle cells. a. pAkt, b. pERK1/2, c. GSK-3β, and d. PRAS40. Control (untreated cells), ZnSO_4_ and insulin treatments are shown. Differences between the expressions of proteins in cells were determined by one-way ANOVA. The results of 3 independent experiments are presented as a mean ± standard error. *p < 0.05, ** *p* < 0.01 and *** *p* <0.001 considered significant when compared to the control.

Densitometry was performed on the protein arrays to determine the level of protein expression between the insulin and ZnSO_4_ treatment and the control. In mouse skeletal muscle cell lines, Akt and ERK1/2 protein expression was significantly higher (*p* < 0.01 and *p* <0.001 respectively) in the ZnSO_4_ treated cells when compared to the control (Fig 6A and 6B, respectively). GSK-3β expression and activation play a critical role in mammalian cells and impacts various cellular processes including glycogen synthesis and glucose transport [30]. The GSK-3β protein levels were significantly higher (*p* <0.001) in cells treated with ZnSO_4_ compared to control (Fig 6C). PRAS40, another important cell signaling molecule, is among the most prominent Akt and mTOR complex 1 (mTORC1) substrates being phosphorylated in response to growth factor stimulation such as insulin in eukaryotes [31]. The PRAS40 protein expression was also significantly higher (*p* <0.001) in mouse cells treated with ZnSO_4_ when compared to control (Fig 6D).

**Fig 7:**
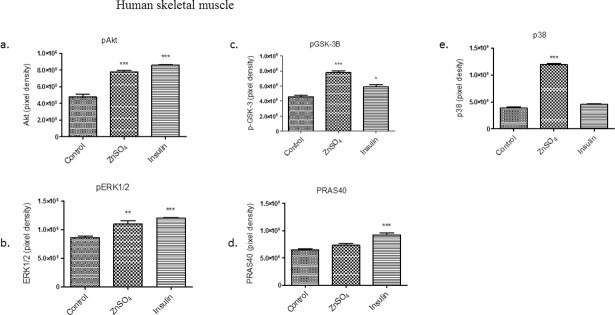
Densitometry results from Fig 5 protein array in human skeletal muscle cells. a. pAkt, b. pERK1/2, c. pGSK-3β, d. PRAS40, and e. p38. Control (untreated cells), ZnSO_4_ and insulin treatments are shown. Differences between the expressions of proteins in cells were determined by one-way ANOVA. The results of 3 independent experiments are presented as a mean ± standard error. * *p* < 0.05, ** *p* < 0.01 and *** *p* <0.001 considered significant when compared to the control.

In the human cell lines, pAkt expression was significantly different (*p* <0.001) over the control during ZnSO_4_ and insulin treatment (Fig 7A). Similarly, pERK1/2 protein expression was significantly different for ZnSO_4_ treatment (*p* <0.01) and insulin treatment (*p* < 0.001) over that of the control (Fig 7B). The GSK-3β protein level was also significantly higher when treated with zinc (*p* < 0.001) than insulin treatment (*p* <0.05) and when compared to the control group (Fig 7C). There was no significant difference in the levels of PRAS40 when treated with ZnSO_4_ and compared to the control (Fig 7D). However, insulin treatment resulted in a significant increase (*p* < 0.001) in PRAS40 compared to the control group. Lastly, ZnSO_4_ treatment resulted in a significant increase in p38 (*p* < 0.001) over the control group but there was no significance identified during the insulin treatment (Fig 7E). p38, is another signaling factor that plays a significant role in the regulation of glucose transport [32].

### Zinc effect of glucose consumption in mouse C2C12 and human skeletal muscle cells

**Fig 8:**
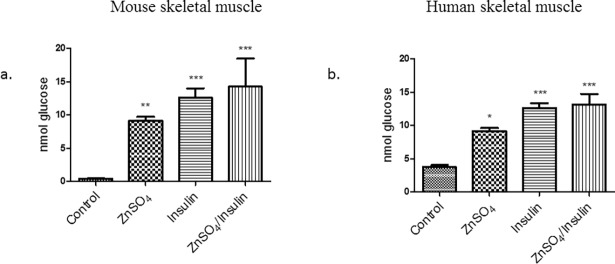
Glucose oxidation assay in the presence of insulin and ZnSO_4_. a. Mouse skeletal muscle cells, and b. Human skeletal muscle cells. The results of 3 independent experiments are presented as a mean ± standard error. * *p* < 0.05, ** *p* < 0.01 and *** *p* <0.001 considered significant compared with the control (untreated cells).

It is clear that zinc affects several cell signaling molecules, analogous to insulin signaling, that are implicated in glucose homeostasis in skeletal muscle. Accordingly, we tested whether zinc could activate glucose oxidation in both mouse and human skeletal muscle cells. Initially we tested glucose oxidation in the presence of insulin treatment, and as expected, insulin significantly induced glucose oxidation in mouse (*p* < 0.001) and human (*p* < 0.001) skeletal muscle cells compared to the untreated cells (Fig 8A and 8B). Similarly, ZnSO_4_ significantly induced glucose oxidation in both mouse (*p* < 0.01) and human (*p* < 0.05) skeletal muscle cells compared to the untreated control (Fig 8A and 8B). We did not observe a significant additive effect of insulin and ZnSO_4_ treatment together when compared to ZnSO_4_ or insulin treatment alone.

### Zinc activation of pAKT potentially acts through a functional insulin receptor

**Fig 9:**
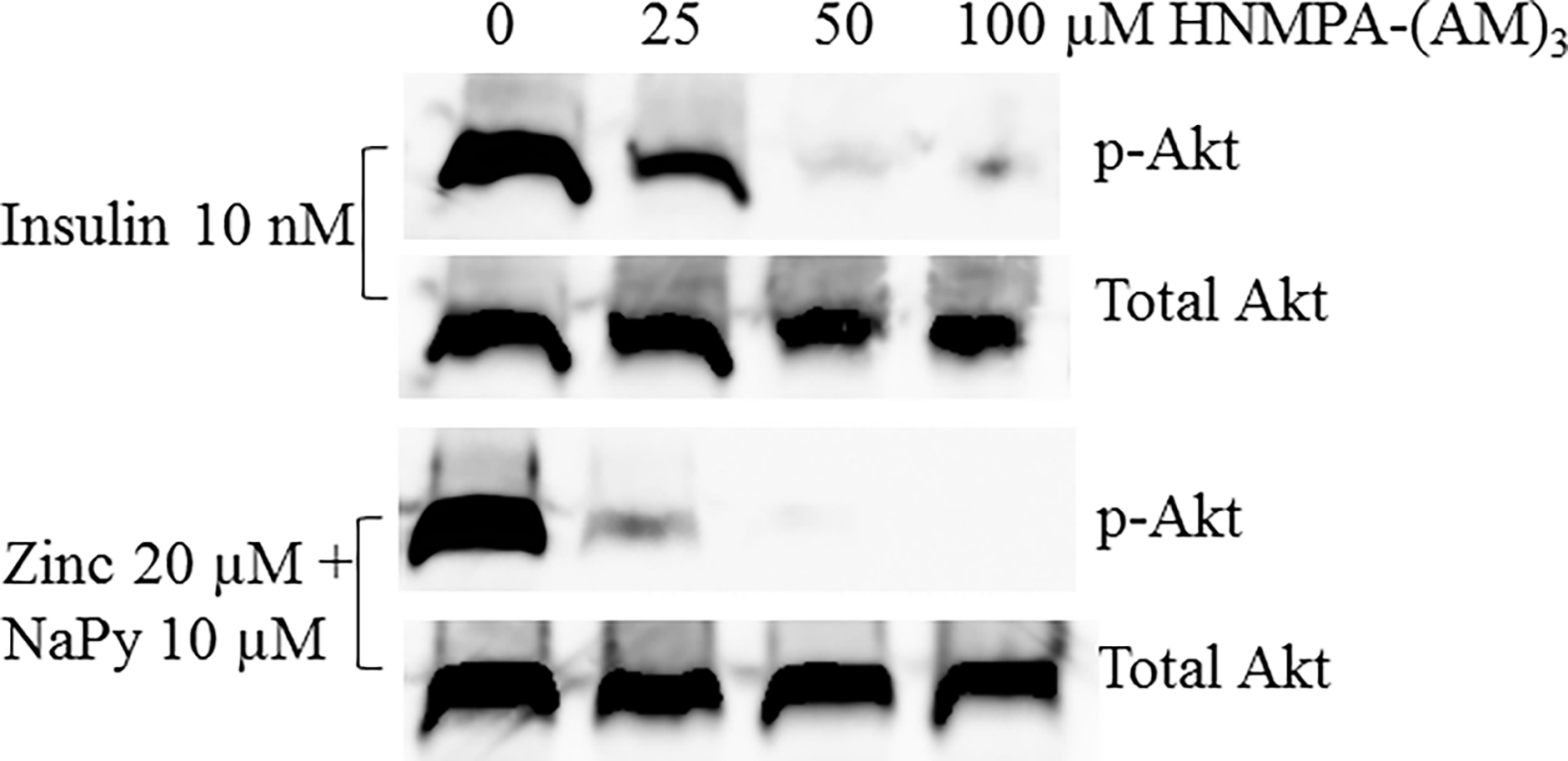
Effect of insulin receptor tyrosine kinase inhibitor HNMPA-(AM)_3_ on insulin and zinc-induced pAkt. Mouse C2C12 skeletal muscle cells were treated with increasing concentrations (0, 25, 50, and 100 μM) of HNMPA-(AM)_3_ followed by either treatment with insulin (10 nM) or zinc (20 μM) for 1 hour. Concentrations of HNMPA-(AM)_3_ are given at 0, 25, 50 and 100 μM. Top panel: insulin-treated measurement of pAkt, Lower panel: zinc-treated measurement of pAkt. Total Akt was used as an internal loading control in both panels and levels of pAKT were normalized to total Akt.

Given that we did not observe an additive effect of insulin and ZnSO_4_ treatment together on glucose oxidation, we sought to determine whether a functional insulin receptor is required to facilitate zinc activation of pAkt. To address this, we utilized C2C12 skeletal muscle cells treated with an insulin receptor tyrosine kinase inhibitor in the presence of insulin or zinc. We observed that 50 μM of the insulin receptor tyrosine kinase inhibitor HNMPA-(AM)_3_ was sufficient to inhibit insulin-induced pAkt (Fig 9). Similarly, we identified that 25 μM of HNMPA-(AM)_3_ inhibited zinc-induced pAkt and this was completely abolished at 50 and 100 μM of HNMPA-(AM)_3_ (Fig 9).

## Discussion

In this study, we analyzed the effects of zinc on insulin signaling in mouse and human skeletal muscle cell lines. Insulin signaling is an important and highly conserved regulatory network coordinating metabolism and growth in multicellular organisms [33]. Dysregulation of insulin signaling is common in metabolic disorders. For example, insulin resistance which is a hallmark of type 2 diabetes mellitus [33], is characterized by a reduced insulin-mediated activation of the PI3K/Akt pathway regulating glucose uptake [34]. Several studies have suggested that zinc, as an essential trace element, has a potent ability on glucose handling by promoting beta cell function and insulin sensitivity [35]. Zinc in skeletal muscle accounts for most of the whole-body zinc (approximately 60%) [36], and as skeletal muscle is the major site of peripheral insulin resistance [37], we investigated the effects of zinc signaling in skeletal muscle cells.

Regarding the participation of zinc in insulin signaling, it is emphasized that this metal ion promotes activation of phosphatidylinositol protein 3-kinase and protein kinase B (Akt), increasing transport of glucose uptake to cells [38]. Our data shows that there is an increase in the phosphorylation of Akt in mouse C2C12 and human skeletal muscle cells treated with zinc, indicating the role of zinc in the induction of Akt phosphorylation. During insulin signaling, the activated insulin receptor kinase, phosphorylates tyrosine residues on insulin receptor substrate 1 (IRS1) and insulin receptor substrate 2 (IRS2) and this subsequently generates a tyrosine phosphorylation cascade to transmit the insulin signal [39]. Our data show that there is an increase in phospho-tyrosine in both mouse and human skeletal muscle cell treated with zinc and suggests that zinc also plays a role in transmitting the insulin signal.

Previous studies have shown that SHP-2 can bind to IRS-1 in response to insulin and modulate receptor tyrosine kinase-mediated signaling [40]. Our data indicates that zinc can also activate SHP-2 in mouse and human skeletal muscle cells. Similarly, studies have shown that the ERK/MAP kinase signaling pathway is an important mediator in insulin responsiveness [41]. According to our results, zinc increases the expression of ERK in both cell lines which might help to improve insulin signaling in skeletal muscle.

Our results have also shown that zinc exhibited insulin-like glucose transporting effects by phosphorylation of key proteins involved in the insulin signaling cascade including GSK-3β. GSK-3β plays several important roles in mammalian cells, and impacts such diverse cellular processes including glycogen synthesis and glucose transport. Overactivity of GSK-3β in skeletal muscle of obese type 2 diabetic humans is associated with an impaired ability of insulin to activate glucose disposal and glycogen synthase [42]. Studies have also demonstrated that inhibition of non-phosphorylated form of GSK-3 can improve insulin-stimulated glucose transport activity through enhanced post-insulin receptor insulin signaling and GLUT-4 glucose transporter translocation [43]. Accordingly, GSK-3β serine phosphorylation, which is the inactive form of GSK-3β, increased after treating cells with zinc.

Our data indicate that zinc increases PRAS40 in mouse skeletal muscle cells. PRAS40 is a regulator of insulin sensitivity which has an inhibitory effect on mTOR and consequently decreases mTOR expression which is a negative regulator of IRS1 in mouse skeletal muscle cell line. It causes an abundance in IRS1 and enhances insulin-mediated Akt phosphorylation and increases glucose uptake [44].

Activation of p38 is not only dependent on stimulus, but on cell type as well. For example, insulin can stimulate p38 in 3T3-L1 adipocytes, but down-regulates p38 activity in chick forebrain neuron cells [45]. We have reported that the expression of p38 increases in human skeletal muscle cells treated with zinc. Although p38 phosphorylation has been reported to be increased in response to insulin in skeletal muscle from non-diabetic subjects [46], we did not observe this in our experiments. It is not clear why p38 did not respond to insulin in this study. Possibly, the thirty-minute, insulin treatment time-point used on the protein arrays missed the maximum response for p38. For example, treatment of mouse L6 myotubes with 100 nM insulin resulted in a maximum p38 response at ten minutes which declined at fifteen minutes of treatment [47]. Similarly, in rat vascular smooth muscle cells (VSMC), p38 was rapidly activated in the presence of insulin at five minutes and quickly declined to basal levels at fifteen minutes [48]. We also identified that zinc can oxidize glucose in both mouse and human skeletal muscle cells. A previous study in rat adipocytes showed that zinc could enhance the metabolism of glucose which was indicative of enhancement of facilitated glucose transport [49]. While both insulin or zinc could independently activate glucose oxidation, an additive effect of insulin and zinc was not observed. Additionally, we found that inhibition of insulin receptor tyrosine kinase activity abolished the ability of insulin and zinc to activate pAkt and therefore suggests that zinc potentially acts through the insulin signaling pathway. These data are supported by previous studies where co-incubation with zinc and insulin did not enhance glucose consumption in normal L6 cells and suggests that zinc and insulin may act through the same downstream pathways [17]. However, in insulin-resistant L6 myotubes, zinc and insulin together could enhance glucose consumption when compared to insulin treatment alone [17]. These data suggest that disrupted insulin signaling in the insulin resistant L6 cells respond differently to zinc than in normal L6 cells and therefore might provide clues on alternative signaling pathways under zinc stimulation.

Skeletal muscle is of major importance in insulin resistance and T2DM as it accounts for 85% of whole body insulin-dependent glucose uptake and therefore plays a critical role in maintaining systemic glucose metabolism [37]. Defects in glucose metabolism in insulin resistant skeletal muscle include reduced insulin receptor tyrosine phosphorylation [50] decreased Akt phosphorylation [51–52], and impaired GLUT4 translocation [52,53]. These insulin protein targets are also activated by zinc treatment in skeletal muscle [3,17,54].

The ability of zinc to regulate insulin signaling processes suggests that this metal ion might have utility to be targeted experimentally to improve the management and/or treatment of insulin resistance. One well-studied target of the insulin-mimetic effects of zinc is protein tyrosine phosphatase 1B (PTP1B). This phosphatase has a key role in the regulation of insulin action by dephosphorylating the insulin receptor and insulin receptor substrates 1 and 2, thereby inhibiting the insulin signaling cascade [55]. It is well-established that zinc can inhibit PTP1B which affects insulin signaling [18]. Moreover, PTP1B knockout mice are highly sensitive to insulin, have low adiposity, and are protected from diet-induced obesity [56]. These studies highlight the dynamic role of zinc in insulin signaling processes and therefore offers opportunities to be further investigated. Type 2 diabetes mellitus is increasing globally and is approaching pandemic levels. Current strategies for prevention are limited in scope and effectiveness, and the persistently high prevalence of T2DM confirm the shortfalls of available therapeutic options. The ‘window of opportunity’ that presents before the loss of β-cell function and the development and progression of T2DM suggests that a strategy aimed at reversing insulin resistance would have major clinical outcomes and benefits for health and wellbeing. From a therapeutic perspective, it is important that the molecular mechanisms of dysfunctional zinc transport and defective zinc compartmentalization are identified in human skeletal muscle cells. Similarly, characterizing how zinc leads to increased glucose homeostasis is paramount to understanding potential intervention strategies where zinc transport could be manipulated experimentally to improve glucose metabolism in patients with insulin resistance or T2DM.

## Conclusions

Zinc is an essential metal ion implicated in several biological processes. Dysfunctional zinc signaling is associated with various disease states. We have shown that zinc independently activates insulin signaling pathway proteins in mouse and human skeletal muscle cell lines. The subsequent phosphorylation events associated with insulin signaling and increased glucose oxidation was also mirrored with zinc treatment leading to the supposition that both insulin and zinc are critically important in maintaining glucose homeostasis in skeletal muscle.

## Supporting information

S1 Dataset Data file of mouse and human protein array densitometry showing the average of three-four independent assays in control, zinc treated, and insulin treated skeletal muscle cells. (XLSX)

S2 Dataset Data file of mouse and human glucose oxidation assay showing the average of three independent assays in control, zinc treated, insulin treated and zinc plus insulin treatment in skeletal muscle cells. (XLSX)
